# ChatGPT in medicine: prospects and challenges: a review article

**DOI:** 10.1097/JS9.0000000000001312

**Published:** 2024-03-19

**Authors:** Songtao Tan, Xin Xin, Di Wu

**Affiliations:** Plastic Surgery Hospital, Chinese Academy of Medical Sciences and Peking Union Medical College, Shijingshan, Beijing, China

**Keywords:** application, challenge, ChatGPT, prospect

## Abstract

It has been a year since the launch of Chat Generator Pre-Trained Transformer (ChatGPT), a generative artificial intelligence (AI) program. The introduction of this cross-generational product initially brought a huge shock to people with its incredible potential and then aroused increasing concerns among people. In the field of medicine, researchers have extensively explored the possible applications of ChatGPT and achieved numerous satisfactory results. However, opportunities and issues always come together. Problems have also been exposed during the applications of ChatGPT, requiring cautious handling, thorough consideration, and further guidelines for safe use. Here, the authors summarized the potential applications of ChatGPT in the medical field, including revolutionizing healthcare consultation, assisting patient management and treatment, transforming medical education, and facilitating clinical research. Meanwhile, the authors also enumerated researchers’ concerns arising along with its broad and satisfactory applications. As it is irreversible that AI will gradually permeate every aspect of modern life, the authors hope that this review can not only promote people’s understanding of the potential applications of ChatGPT in the future but also remind them to be more cautious about this “Pandora’s Box” in the medical field. It is necessary to establish normative guidelines for its safe use in the medical field as soon as possible.

## Introduction

Highlights

- ChatGPT has left a profound impact on people because of its incredible potential.
- Artificial intelligence will inevitably infiltrate and change every facet of contemporary life.
- ChatGPT has successfully applied itself in numerous aspects of medicine.
- ChatGPT’s applications in clinical practice and medical research face limitations
- We should be more cautious about the applications of this “Blind Box” in medicine.

In November 2022, OpenAI (San Francisco, CA, USA) launched a product of great significance called the Chat Generator Pre-Trained Transformer (ChatGPT)^1^. As a large language model combining artificial intelligence (AI) and natural language processing, ChatGPT has the ability to comprehend conversations and then respond with human-like, high-quality texts^2^. With the ability to process large volumes of data rapidly and precisely, ChatGPT can be applied in different fields, such as translation, text summarization, programming and so on^3^. Subsequently, a renewal version with a more powerful capability named GPT-4.0 was published. Just 4 months after its introduction, ChatGPT has amassed more than one billion monthly users, demonstrating its vast popularity.

Similarly, ChatGPT has also attracted a great deal of interest from the medical community for its potential to improve efficiency in clinical practice and accelerate medical research^4^. Numerous efforts have been undertaken to investigate the potential applications of ChatGPT in the medical domain, and many positive outcomes have been obtained. However, a variety of concerns about plagiarism in academic writing, the dissemination of misinformation, and the risk of clinical application have also been raised.

In this review, we hope to summarize both promising applications and worrying challenges of ChatGPT in the medical domain and provide researchers with a comprehensive understanding of ChatGPT to better utilize this technology.

## ChatGPT in medicine: prospects

### Revolutionizing healthcare consultation

Leveraging the rapid processing of substantial datasets, ChatGPT can expeditiously generate content pertaining to specific diseases^5^. Through the utilization of ChatGPT, patients can effectively communicate with their physicians by having a preliminary understanding of their condition before consultation.

As shown in the study by Endo and colleagues., the majority of answers generated by ChatGPT for liver transplantation received high praise from experts. Specifically, more than 70% of the responses received ratings of “very good” or “excellent”. Most of the interviewed experts believed that patients could utilize ChatGPT as an initial resource to address uncertainties^6^. Research in periodontal disease, breast reconstruction, otolaryngology, dermatology and other areas has also shown that ChatGPT is adept at addressing patients’ concerns^7–10^.

Compared to traditional search engines, ChatGPT provides more comprehensive, specific, and scientific answers. Choo and colleagues conducted a study on breast implant-associated anaplastic large-cell lymphoma to assess the quality of answers from ChatGPT and Google^11^. According to the feedback from five experienced breast augmentation surgeons, ChatGPT outperformed Google in providing high-quality answers to breast augmentation. Meanwhile, ChatGPT also reminded patients that they should consult with their healthcare teams for further treatment^12^.

Notably, ChatGPT can emulate everyday conversations and exhibit empathy, thereby enhancing the accessibility of responses^13^. Upon comparing responses from doctors with those from ChatGPT, Ayers *et al.* ^14^ also observed that ChatGPT generated responses of high quality. The proportion of responses rated as empathic or very empathic was greater for ChatGPT than for doctors. Additionally, 78.6% of the population showed a greater inclination to accept responses from ChatGPT.

Moreover, ChatGPT possesses significant advantages in the privacy-conscious field of mental disorders. ChatGPT can eliminate the impact of clinicians’ accents and facial expressions on patients during consultations, enabling broad, consistent and objective screening of mental disorders. From the perspective of patients, they can obtain health information related to mental disorders in a more private environment, thereby helping them solve issues related to stigma^15^. Additionally, ChatGPT can be used to describe and identify emotions from behavioural descriptions in various scenarios and then abstract emotional states in a profound, multidimensional, and integrated manner. This suggests that ChatGPT may be employed for consciousness training in patients with impaired emotional awareness and may be beneficial for psychiatric diagnosis and evaluation^16^.

Through ChatGPT, patients can transcend the constraints of geographical location or objective conditions to access high-quality medical information at any time and place, thereby significantly mitigating disparities in healthcare resources acquisition. For example, the research by Choudhary *et al.* ^17^ suggested that ChatGPT may have a number of advantageous applications in travel medicine, including providing real-time updates on disease outbreaks and health risks, personalized vaccination and prophylactic medication recommendations, and practical advice for staying healthy during travel. These capabilities not only prepare travellers to avoid potential health risks but also offer medical professionals a valuable resource to serve patients more efficiently and effectively, ensuring travellers receive optimal advice and support.

### Assisting patient management and treatment

In addition to offering powerful assistance and support to patients, ChatGPT can also aid doctors in patient management and treatment.

ChatGPT has demonstrated outstanding performance in various clinical applications. For instance, in the Specialty Certificate Examination in Dermatology, ChatGPT achieved an accuracy of 80%, far exceeding the 60% accuracy required by the test^18^. In the more intricate treatment of fungal diseases, ChatGPT continued to provide satisfactory responses in various aspects, including diagnosis, auxiliary examinations, treatment, and rational medication^19^. Even in cases necessitating multidisciplinary treatment (MDT) discussions, the diagnostic and therapeutic suggestions given by ChatGPT aligned well with the management recommendations from experts^20^. Therefore, researchers believe that ChatGPT can be used to analyze medical records, literature, and the latest clinical guidelines to generate summary texts. These texts can be further evaluated, organized, and utilized by experienced clinicians to support clinical decision-making and ultimately provide personalized treatment options for patients^21^.

Moreover, ChatGPT's robust image processing capability is impressive^22^. With this ability, ChatGPT can aid clinicians in surgical oncology to obtain a clearer understanding of tumour location or anatomic abnormalities and facilitate a more comprehensive evaluation of tumour resectability, the extent of resection and surgical methods^23^. Besides, through the analysis of a broad range of images, skin lesions can be examined, retinopathies can be detected, and diseases can be identified according to pathological sections of tissue samples^24^. Simultaneously, personalized images can be generated according to patients’ descriptions, allowing them to preview the expected postoperative outcome, which is especially helpful for doctor–patient communication in the field of plastic surgery. By concretizing patients’ expectations, ChatGPT can also help doctors design more personalized surgeries and alleviate the preoperative anxiety of patients^25^.

Furthermore, ChatGPT's powerful conversational ability can also play an important role in clinical practice. The tool can be developed into a chatbot to assist doctors in triage by providing a preliminary classification of their condition and urgency. It was found that ChatGPT could quickly categorize patients with multiple traumas and assess patients in need of urgent intervention and care^26^.

Apart from helping doctors in clinical diagnosis and treatment, ChatGPT also exhibits remarkable proficiency in clinical paperwork. Researchers have found that ChatGPT could extract pertinent information from medical records and generate a coherent narrative, which could help doctors complete patient notes, operation records and related medical paperwork more efficiently^24,27,28^.

### Transforming learning for novice clinicians and students

ChatGPT can be incorporated into the present education platform as a virtual tutor, creating a more engaging and impactful learning environment tailored to student’s individual learning pace and preferences^29^. It can provide a wealth of relevant information that enhances students’ deeper understanding of medical knowledge and further stimulates their critical thinking ability^30^.

For example, practice has shown that using ChatGPT as an educational tool in a public health course offered significant assistance in addressing intricate medical questions pertaining to vaccination. Students have expressed considerable satisfaction with this educational encounter and articulated a keen inclination for the next engagement^31^. Besides, the impressive programming capabilities of ChatGPT render it an appealing tool for fostering education in bioinformatics data analysis for beginners. It can not only assist students in the initial writing of the code but also help them rectify errors^32^. Furthermore, Choudhary *et al.* ^33^ discovered that incorporating ChatGPT into veterinary anatomy education presented numerous benefits^33^. Serving as a virtual tutor, ChatGPT is capable of providing personalized and interactive learning experiences tailored to the unique needs of each student. Its ability to deliver detailed anatomical information, facilitate discussions on comparative anatomy, and support case-based learning significantly improved students’ comprehension. This integration not only makes the educational content more accessible but also enriches the learning environment by introducing a variety of innovative approaches to the study of anatomy, thus complementing traditional teaching methods.

Given the excellent performance of ChatGPT in various medical professional qualification exams, researchers believe that it could function as a supplementary resource aiding medical students in their preparation for the qualification examinations^34^. Students may ask questions about specific medical topics to ChatGPT and then receive precise replies that help them better comprehend the material^35^. Moreover, ChatGPT can generate detailed case scenarios and produce high-quality exam questions to evaluate the actual level of students. For novice surgeons requiring clinical experience, ChatGPT can simulate surgery procedures, delineate the surgical steps, and elucidate the rationale behind each action, which can help them become familiar with these procedures before the actual operation^27^. Irrespective of regional or institutional variations, clinicians can access uniform and high-quality visual resources for learning and training^25^.

### Facilitating clinical research

Deeper research on unexplained clinical problems can not only benefit patients but also promote the iterative updating of medical knowledge. ChatGPT can help doctors resolve problems encountered in clinical practice and form research ideas. Nachalon and colleagues used ChatGPT to generate research ideas on dysphagia, including the use of teletherapy, the impact of comorbidities on dysphagia, and different clinical interventions. Experts in this field believed that the generated research ideas were feasible, novel and clinically meaningful^36^.

Additionally, ChatGPT can also help improve research efficiency^37^. ChatGPT has the powerful ability to collect and summarize information, which can help researchers keep abreast of the latest progression in related fields. Besides, based on its robust data analysis capability, ChatGPT can help researchers cope with tasks involving large amounts of biological data more easily. Chen *et al.* ^38^ used ChatGPT to process and analyze large-scale gene expression data and constructed GenePT to infer gene function and interrelationships from the gene expression profiles of millions of cells. In addition to predicting gene relationships, ChatGPT can also be used to explore molecular interactions and predict possible drug targets^19,39^.

Finally, ChatGPT has performed excellently in article writing and editing. Articles generated by ChatGPT have been found to obtain higher scores than those written by humans. Its writing is highly standardized and structured and is also logical^40^. For example, ChatGPT was asked to write a short article on how homocysteine-induced osteoporosis. It presented a satisfactory answer with an organized structure, indicating its auxiliary role in article writing^41,42^. Moreover, for researchers who are not native English speakers, the use of words always appears to be less authentic, and grammar or vocabulary errors may inadvertently occur. The advent of ChatGPT seems to be a great solution to this problem. In a test, ChatGPT successfully identified 86 out of 171 errors that the editor had found and then provided appropriate revision suggestions for 72 of them, which implied that ChatGPT might be a valuable tool for editing and polishing manuscripts^43^.

## ChatGPT in medicine: challenges

Although ChatGPT has achieved satisfactory results in an increasing number of application trials, related concerns and criticisms have also emerged. Out of the 60 publications included in a systematic review by Sallam *et al.* ^44^, 58 (96.7%) articles expressed concerns regarding the use of ChatGPT. Specifically, these concerns include research fraud, lack of originality, ethics, copyright, legal difficulties, hallucination brought about by inaccurate content, risk of prejudice, risk of information leakage.

### The risk of research fraud in academic writing

The possibility of research fraud associated with utilizing ChatGPT to produce academic publications, such as plagiarism, ghostwriting or falsified research, has become the primary concern^45^. On the one hand, ChatGPT is indeed valuable for helping with data analysis and improving writing efficiency. On the other hand, its capacity to quickly generate texts is so powerful that it may also facilitate academic fraud by creating bogus material that is hard to spot.

However, at present, the efficiency of AI detectors is relatively low, and determining the difference between writing from AI origin and writing created by humans is challenging. According to the study by Odri *et al.* ^46^ on detecting generative AI in scientific articles, the majority of AI detectors failed to identify texts produced by generative AI, and even some of the human-written texts were mistakenly recognized as being generated from AI. In addition, the authors identified a number of methods to avoid AI detection, which might be simply obtained and applied to “improve” documents originating from AI. In the future, it is possible that frequent reading of texts from AI origin might lead people to subconsciously write in an AI-like manner, which may further increase the difficulty of distinction. Additionally, due to ongoing upgrades to generative AI, such as the introduction of GPT-4.0, AI detectors will become nearly useless, and the academic community might be overrun by undetected literature written by ChatGPT.

Furthermore, the broad application of ChatGPT in research may impede critical thinking and creativity. Under no circumstances can we deny that human judgment and critical thinking are crucial for guaranteeing the validity and comprehensiveness of research discoveries. Rather than relying exclusively on ChatGPT, we ought to integrate human expertise with its immense potential^2^.

### Ethics concerns in academic writing

Whether ChatGPT can be listed as a co-author of publications has always been a controversial issue. In a commentary by Marchandot and colleagues, ChatGPT was applied to help with the writing and editing process, which significantly enhanced writing efficiency. Therefore, the authors believed that it was appropriate to credit ChatGPT as a co-author or even the primary author of their publication^47^. In fact, ChatGPT has already been listed as a co-author in several works before it sparked a heated debate^48,49^. However, it appears that the academic community has not embraced this practice, as indicated by the lack of follow-up publications titled ChatGPT as a co-author.

Currently, due to ChatGPT’s inability to take responsibility for its contents, it seems unacceptable to list ChatGPT as an author according to the guidelines of the International Committee of Medical Journal Editors (ICMJE)^44,50^. The majority of publishers and journals also oppose this practice, with some of them outright forbidding any use of content (texts, figures, images and so on) generated by AI in publications^51,52^.

It is urgent and essential to establish policies and guidelines for the application of ChatGPT in scientific research, as the application of generative AI in scientific research has become an inevitable trend.

### AI hallucination brought by inaccurate content

Although ChatGPT’s responses are often accurate, there are instances where they seem reasonable at first but are actually incorrect or even ridiculous. A phenomenon termed AI hallucination occurs when AI presents a seemingly credible answer that is wholly made up^53^. Although the frequency of AI hallucination may actually be low, it still substantially erodes users’ faith in ChatGPT. Therefore, it must be recognized that blindly trusting ChatGPT may have a negative impact on our judgment and decision.

The most criticized aspect of AI hallucination is the phenomenon of ChatGPT, which provides faulty or nonexistent scientific references. According to the study by Frosolini *et al.* ^54^, out of the 120 references provided by ChatGPT 3.5, only 16.66% of the references were completely correct, and most of them were nonexistent or inaccurate, with various errors. Although ChatGPT 4.0 outperformed version 3.5 in terms of reference accuracy, more than 25% of the references provided by version 4.0 were still determined to be incorrect. Similar results have also been reported in other studies^55–57^.

In fact, the errors in the references provided by ChatGPT are quite covert based on our experience. Although a straightforward search for these titles yields no results, we do find some actual articles with similar titles. Moreover, ChatGPT does not create fictitious authors from thin air; in contrast, the “authors” of these fictional references are real researchers in certain fields. For the references with multiple “authors”, these scholars are not entirely unrelated, as they have indeed collaborated to publish articles. To some extent, ChatGPT is also smart and intelligent in terms of fabrication.

This matter warrants particular attention since AI hallucinations may impair researchers’ perception and further skew their findings. Therefore, some scholars do not encourage the use of ChatGPT in scientific research. At least, any content generated by ChatGPT should be carefully examined before being included in the manuscript, even if it is correct and efficient at most of the time.

### The risk of using ChatGPT in clinical practice

It appears that there is still a long way to go before ChatGPT can be truly implemented in clinical practice, although it offers enormous prospects for addressing the issue of unequal distribution of medical resources in theory. In contrast to medical education and research, precision is extremely important in clinical practice, as any minor mistake may lead to irreversible consequences for patients.

Reproducibility is a major issue since ChatGPT may give different responses to marginally different descriptions of the same question^44^. Furthermore, due to the large gap in economic status and medical resources, treatment options may differ between regions, even for the same disease. However, ChatGPT’s responses are often superficial and lack specificity, making it difficult to provide professional support for clinicians, even though they are appropriate for nonmedical individuals as a popularization of medical information^58,59^. According to the study by Seth *et al.* ^60^, although ChatGPT successfully made the correct diagnosis and proposed several therapies, it could only provide general comments on each management option and was unable to identify specific indicators that determined each option to be appropriate.

In the future, if ChatGPT could be applied in clinical practice, access to real but sensitive patient data will be required by the AI system for the greatest accuracy and utility^2^. Then, concerns about the safety of sensitive information and the privacy of patients emerge. How can we make sure that unauthorized access and data leakage will not occur during this process, which may seriously violate patient privacy and trust^61^. Currently, there is still a lack of supervision and standardization of the responsibility system for ChatGPT. Therefore, it is imperative to establish pertinent ethical guidelines and regulations as soon as possible to ensure the safe and responsible use of ChatGPT in clinical practice^59^.

## Discussion

Since its inception, ChatGPT has aroused great interest among people. The beautiful prospect of AI integrating into and changing modern life in the future seems to be presented. In the past year, researchers have made various attempts to explore the possible applications of ChatGPT in the medical field, with clinical practice seeing the most of these efforts. People are constantly amazed by one satisfactory result after another and the incredible potential of ChatGPT. The emergence of ChatGPT seems to be a great tool to help improve the efficiency and quality of clinical work, as well as partially solve the major problem of uneven distribution of medical resources. In the future, we may also witness some significant changes that ChatGPT will bring to healthcare consultation, medical education, and clinical research, which will become a reality one by one.

Nevertheless, it should be clearly noted that the vast majority of official journals, publishers, and institutions still hold a conservative attitude towards the application of ChatGPT and carefully restrict its use in scientific research. Although the promising application prospects of ChatGPT are highly anticipated, based on our limited understanding, we think that the current application foundation of ChatGPT in clinical practice (requiring 100% accuracy) and medical research (requiring authenticity and rigour) is still immature, at least before the publication of an authoritative normative guideline. Additionally, we would like to remind the public that even though ChatGPT has demonstrated capabilities far beyond traditional search engines in providing medical consultations, the fundamental issue of not being able to guarantee 100% accuracy of recommendations and not being able to take responsibility still remains unchanged. We should never regard ChatGPT as a substitute for professional doctors. However, the application of ChatGPT in medical education is relatively safe and deserves continuous exploration. Perhaps we should embrace the changes in medical education brought by ChatGPT with a more open attitude and actively integrate it into our life-long medical learning.

## Ethical approval

No ethical approval is needed for this study.

## Source of funding

This study is supported by Medical and Health Technology Innovation Project of the Chinese Academy of Medical Sciences (2021-I2M-1-052).

## Author contribution

S.T. and X.X. contributed equally to the conceptualization and writing of the manuscript. D.W. was involved in the critical review and revision of the manuscript for important intellectual content.

## Conflicts of interest disclosure

None.

## Guarantor

Di Wu.

## Data statement

Data sharing is not applicable to this article as no new data were created or analyzed in this study.
